# Featured Abstracts from the 18th National Conference on Chronic Disease Prevention and Control

**Published:** 2004-03-15

The objective of this state-community partnership initiative was to mobilize community action to eliminate health disparities through coalition-driven, asset-based, neighborhood-specific program designs. Participants included 16 African American, Hispanic, Asian American, and other underserved neighborhoods throughout New York State.

From April 2000 through March 2003, the New York State Department of Health funded the Minority Health Community Partnerships initiative, establishing 16 coalitions to address health disparities. Coalitions were funded for 3 years and received training on coalition development and the asset-based community development model. Interventions were designed around the strengths and resources of coalition members and community assets — individuals, associations, and institutions. Disparities addressed included asthma, cardiovascular disease, diabetes, HIV/AIDS, oral health, and access to care. Peer education, provider education, case management, media messages, and community-wide outreach strategies were used. Success was measured based on the extent to which intervention objectives were met, community assets utilized, and coalition members engaged.

A total of 400 organizations (e.g., faith-based, educational, health care, commercial, financial, media) and residents engaged in the process. Three hundred peer educators were trained to deliver prevention messages, and 300 providers were trained on prevention strategies. A total of 60,000 community residents were reached with prevention messages. A total of $2 million in additional funding was leveraged.

A coalition-driven, asset-based approach to addressing health disparities creates opportunities for underserved populations to make collective decisions about community health and to implement strategies that build skill sets and competencies useful to the community.

