# Supplementary material for: Featured Abstracts from the 18th National Conference on Chronic Disease Prevention and Control
Source: Prev Chronic Dis. 2004 Mar 15;1(2):A09. (PMC1189735)
Supplement: Related texts

# Sub article: Implementation of the Coordinated Approach to Child Health (CATCH) Program in Texas

**Published:** 2004-03-15

**Authors:** Cristina Barroso, DM Hoelscher, SH Kelder, C McCullum, JL Ward, P Cribb, N Murray

**Affiliations:** University of Texas-Houston School of Public Health, Center for Health Promotion and Prevention Research

Implementation of the Coordinated Approach to Child Health (CATCH) program was evaluated by child nutrition (CN) and physical education (PE) specialists who attended CATCH trainings in Texas during 2001 and 2002.

Coordinated school health programs offer an effective means of providing consistent health promotion messages. CATCH is a school-based nutrition and physical activity program designed to reduce risk factors — unhealthy dietary intake, physical inactivity, and smoking, for example — for chronic diseases among elementary school children.

CN and PE specialists who attended trainings completed a mail survey that assessed factors influencing implementation of CATCH at their schools. A cross-sectional study design was used: response rates were 38.7% for PE specialists and 39.9% for CN specialists.

The mean score for the percentage of PE lessons using CATCH PE activities was 44.7%, and the mean score for the percentage of CN specialists implementing CATCH Eat Smart guidelines was 80.4%. Likert scales were used to score satisfaction with the CATCH program, where 1 = strongly disagree and 5 = strongly agree, and mean scores were calculated. Both PE and CN specialists reported that the CATCH program helped them meet PE and food service goals (mean of 3.8 for each). PE and CN specialists were satisfied with the CATCH program (mean scores were 4.1 for PE specialists and 3.9 for CN specialists). PE and CN specialists would recommend the CATCH program to others (mean of 4.1 for each).

Results indicate that CATCH is being implemented and is viewed positively by both CN and PE specialists in schools in which personnel were trained using a coordinated school health model.

# Sub article: Identifying Walking and Trail Use Supports and Barriers Through Focus-Group Research

**Published:** 2004-03-15

**Authors:** Ericka Burroughs, RM Fields, ML Granner, PA Sharpe

**Affiliations:** University of South Carolina, Arnold School of Public Health, Prevention Research Center

Walking and trail use supports and barriers in a South Carolina county were identified. As part of a community-based participatory research project, focus groups were conducted to develop social marketing activities.

Twelve focus groups were conducted to identify themes related to physical activity, walking, and trail use. Questions covered preferred walking location, social support for physical activity, preferred incentives, and characteristics of trails. Discussions were recorded, transcribed verbatim, and analyzed using NVivo software.

Results from the focus groups revealed that, while concern for safety was the primary walking barrier cited by women and older adults, walking groups were a potential support for them. There were differences in preferences between active and inactive groups and male and female participants. The secluded nature of some trails had positive and negative aspects. Furthermore, awareness of existing trails was low..

This focus-group research indicated that the social marketing intervention in this community should address safety concerns and emphasize walking groups for women and older adults. The lack of awareness of available walking trails indicated a need to publicize trails as part of the intervention.

# Sub article: Moving Right Along: A Creative Partnership to Engage Older Adults in Physical Activity and Nutrition Programs

**Published:** 2004-03-15

**Authors:** Denise Chapel, MM McCulla, B Reinsch, C Warren

**Affiliations:** Community Nutrition, Missouri Department of Health and Senior Services

A creative partnership was established among Tri-Parish Nursing Ministries, The Arthritis Foundation, Missouri Extension Services, and the Saint Louis County Department of Health to promote quality of life for older adults through physical activity and health education messages.

Twenty-nine participants met twice a week in a local church to exercise for one hour with a trainer from PACE, or People with Arthritis Can Exercise, and then to receive 30 minutes of nutrition education from a Saint Louis County registered dietitian using the Missouri Extension's Health for Everybody program.

A pilot study design was used to engage Saint Louis North County older adults in a physical activity program. Participants were recruited from doctors' offices, local churches, grocery stores, and libraries, using flyers and brochures. Newspaper ads were also used. Outcomes desired included a minimum participation rate of 50% at the end of the 6-week sessions, increased flexibility, and positive feelings reported in Health for Everybody evaluation forms.

The participation rate for the program was 62% at the end of 6 weeks. Mean age of participants was 74 years. All participants reported positive feedback, indicating that they enjoyed sessions and "learned something new." Mean Healthy Eating Index score was 68.6. Feedback revealed that participants desired more ways to exercise outside of class, so Tai Chi instruction and pedometers will be incorporated into future programs.

Nutrition education and structured exercise classes in a safe setting, tailored to varying levels of ability, engaged older adults for the 6-week session.

# Sub article: Improving Care for the Homeless Population Using the Chronic Care Model

**Published:** 2004-03-15

**Authors:** Bechara Choucair, T Palmer

**Affiliations:** Health Care for the Homeless, Medical Director, Crusader Central Clinic Association

The objective of this study was to adapt the chronic care model to improve outcomes among the homeless population in the Health Care for the Homeless Program at Crusader Clinic in Rockford, Ill.

A major goal of *Healthy People 2010* is to eliminate health disparities. Obtaining baseline data to assess clinical quality is a necessary step toward identifying areas for eliminating health disparities between the homeless population and the general population.

Diabetes, hypertension, and asthma were the chronic conditions chosen for study because of their prevalence among the Rockford, Ill, homeless population. Staff members were divided into 3 teams, each addressing one of the chronic conditions. A registry was established to track the outcome of each condition. The Patient Electronic Care System (PECS), provided by the Health Disparities Collaborative, was used to track outcomes.

The number of hypertension patients with a blood pressure of less than 140/90 mm Hg increased from a baseline of 31% to 48%, despite the addition of newly diagnosed patients. Homeless clients with diabetes had an average HbA1c of 7.9%. Among asthma patients, 25% had a severity assessment documented, and 50% had received an influenza vaccine.

Using the chronic care model and intensive follow-up, improvements in outcomes can be significant, despite many barriers to optimal care among the homeless population.

# Sub article: Utah K-8th Grade Height and Weight Measurement Project

**Published:** 2004-03-15

**Authors:** Karen Coats, MD Friedrichs, JL Ware

**Affiliations:** Utah Department of Health, Heart Disease and Stroke Prevention Program

Baseline height and weight data were established on more than 10,000 Utah children in kindergarten through eighth grade.

The prevalence of childhood overweight in the United States has tripled since 1966. In Utah, the Behavioral Risk Factor Surveillance System is used to track adult overweight and obesity, and the Youth Risk Behavior Survey is used to track adolescent overweight. However, no data source exists to track childhood overweight.

Height and weight data were collected on 10,041 students at randomly selected elementary and middle schools in Utah. School nurses assisted with recruiting volunteers, scheduling, and measuring. The Heart Disease and Stroke Prevention Program provided staff, equipment, and measurement protocol. Screens were used to protect children's privacy. Gender, birth date, height, and weight were recorded.

One fourth of Utah students in kindergarten through eighth grade — 28% of boys and 23% of girls — were overweight or at risk of becoming overweight. The percentage of students considered overweight was 12.2% — 14% for boys and 10% for girls. Since 1993, overweight among third-grade boys has increased by 119%; overweight among third-grade girls has increased by 40%.

Utah mirrors the national trend of increasing rates of childhood overweight; therefore, surveillance of kindergarten through eighth-grade students is important. With assistance from school nurses and parent and teacher volunteers, data can be collected in a timely manner. Consistent methodology is recommended, and children's privacy should be considered.

# Sub article: BC Walks: The Use of Mass Media and Community Programming to Promote Walking

**Published:** 2004-03-15

**Authors:** Patricia Fell, B Fisher, B Reger, D Spicer

**Affiliations:** Community Health Services, United Health Services Hospitals, Wilson Medical Center

The objective of this study was to increase the number of people who meet the recommended standard for moderate-intensity physical activity through walking and to assess the use of mass media and community programming to increase walking awareness and behaviors in individuals. The goal was to have 10,000 people pledge to start walking 10 minutes a day, working up to 30 minutes a day.

*BC Walks* media campaign, public relations, and public health activities were conducted in Broome County, NY, during May and June 2003. Broome County's total population is 200,500.

*BC Walks* promoted walking among the residents of Broome County through a mass media campaign including television, radio, cable, and print. Additional programming included walking events such as "Walk With a Doc" and a walk with a county executive downtown at lunchtime. Preprinted prescription pads for walking were given out by health care providers to their patients. A speakers' bureau was developed to provide speakers for service groups, a program that reached more than 1000 people with the *BC Walks* message. Additionally, a Web site was developed for individuals to log their minutes and see the latest activities of *BC Walks*. Worksite challenges using pedometers as incentives were conducted in 30 businesses representing more than 4000 employees. Five schools introduced programs that encouraged walking among students, staff, and parents. Community programs on walkable communities were conducted in 4 local municipalities.

During the 2-month program, 10,800 people signed pledge cards to start walking 30 minutes a day. This mass media and community program demonstrated an increase in the number of people who pledged to meet the recommended standard for moderate physical activity.

# Sub article: Using CDC’s *School Health Index* to Improve the Physical Activity and Nutrition Environments in 15 Michigan Public Schools

**Published:** 2004-03-15

**Authors:** Nicholas Drzal, L Grost, E Coke-Haller, A Murphy

**Affiliations:** Michigan Department of Education, Office of School Excellence

The childhood obesity crisis was addressed in Michigan by implementing the Centers for Disease Control and Prevention's *SHI: School Health Index* in 15 schools during the 2002–2003 school year.

The Michigan departments of education and community health have partnered to encourage schools to improve their physical activity and nutrition environments by using the *School Health Index*. Fifteen Michigan public schools agreed to implement School Health Index and report their results during the 2002–2003 school year in return for $1000.

Each school was required to form a Coordinated School Health Team (CSHT), designate a team leader (on-site coordinator), and work with a trained *School Health Index* implementation facilitator. Facilitators assisted the on-site coordinator and CSHT through *School Health Index* implementation.

Evaluation results indicated that using the *School Health Index* encouraged schools to create and maintain building-level CSHTs, increased on-site coordinators' familiarity with physical activity and nutrition policies, and encouraged physical activity and nutrition promotion activities. Moreover, this process increased staff and student opportunities to be physically active and, in addition, increased the number of nutrition learning opportunities during school hours. Despite these successes, some schools experienced difficulty maintaining their teams over the year and lacked administrative support.

In summary, the *School Health Index* is a valuable, free tool for a committed school staff member or public health agency representative to use in mobilizing a school to offer more physical activity and nutrition education opportunities and to serve healthier food.

# Sub article: Healthy Weight in Schools: Supporting Schools Through Partnerships

**Published:** 2004-03-15

**Authors:** Shannon Carney Oleksyk, EC Haller

**Affiliations:** Michigan Department of Community Health, Division of Chronic Disease and Injury Control/Cardiovascular Health, Nutrition & Physical Activity

In 2002, statewide consensus recommendations — *The Role of Michigan Schools in Promoting Healthy Weight* — were developed under the leadership of the state departments of education and public health. Thirty statewide organizations participated in the consensus process. Many of these original partners and additional new organizations are actively collaborating to identify and implement initiatives to help schools implement the consensus guidelines.

Five specific initiatives have evolved: 1) a development, printing, and distribution plan for family education materials; 2) grants to schools to implement the Centers for Disease Control and Prevention's *SHI: School Health Index* self-assessment and planning tool and the U.S. Department of Agriculture's *Changing the Scene* kit; 3) a Web site for easy access to resources and assessment tools; 4) a statewide conference on healthy school environments; and 5) leadership and development of resources for the *Michigan Action for Healthy Kids Coalition*.

Preliminary evaluation results suggest that schools find these tools useful in creating healthy school environments and promoting healthy weight.

Schools are struggling to address obesity during a time of limited resources. It is imperative that states do everything possible to help them in their efforts. Partnerships reduce duplication and produce innovative products and tools.

# Sub article: SHI: School Health Index: Implementing Changes in the Third Edition

**Published:** 2004-03-15

**Authors:** Samantha Harrykissoon, H Wechsler

**Affiliations:** Centers for Disease Control and Prevention, Research Application Branch/Division of Adolescent and School Health

The Centers for Disease Control and Prevention's *SHI: School Health Index: A Self-Assessment and Planning Guide* is designed to help schools assess and improve their physical activity, healthy eating, tobacco use, and unintentional injury and violence prevention policies and programs in the context of a coordinated school health program.

In addition to reviewing the history, purposes, and structure of the *School Health Index*, contributors to the guide describe the process of developing the third edition and identify changes that have been made to the new edition, which will be released in 2004. Changes include the addition of items for assessing a school's injury prevention policies and programs and the revision of other items based on feedback from public health practitioners. The third edition also will feature a new interactive on-line version that allows users to tailor the *School Health Index* according to health and safety topics — physical activity, nutrition, tobacco-use prevention, and injury and violence prevention, for example.

The presenters share information collected from education agencies, health departments, schools, and other programs across the nation about how they used the *School Health Index*, including descriptions of innovative strategies for promoting its use and increasing its impact.

Contributors to the guide will continue their interactive tradition by encouraging *School Health Index* users to share their experiences in using or promoting the *School Health Index*, as well as to suggest ideas for improving the guide or maximizing its effectiveness.

# Sub article: Policy and Environmental Change Strategies to Reduce Obesity: Action Packets

**Published:** 2004-03-15

**Authors:** Lori Kaley, D Wigand, K Whalen, A Root>

**Affiliations:** Coordinator, Community Health Initiatives, University of Southern Maine, Edmund S. Muskie School of Public Service, Institute for Public Sector Innovation

Action packets were developed to help communities, schools, and work sites in Maine develop policies and produce changes in the environment that would lead to improved nutrition and increased physical activity.

The Maine Cardiovascular Health Program and the Maine Nutrition Network collaborated to develop policy and environmental change strategies with a focus on increasing physical activity and improving nutrition. Evidence-based findings and the social/ecological model were used in determining strategies. Strategies were presented to state- and community-level stakeholders. Action packets are being used to implement strategies.

A concept and framework development team, action packet workgroups, and potential users collaborated to produce action packets. Two action packets are *Promote Trail Development and Use of Safe Community Routes for Walking and Biking* and *Develop Policies that Support Healthy Eating at Group Events*. Two additional action packets in production are *Enhance Access to Places for Physical Activity* and *Develop Policies that Support Health Options in Vending Machines*. Action packets include case studies, action steps (including outcome evaluation), advocacy materials, Web resources, and references.

Regional action packet educational sessions were provided. An educational session evaluation report indicated that a majority of participants would incorporate strategies into their action plans. Hard copies of action packets have been distributed and are also available on-line. Ongoing process evaluation is being completed.

Policy and environmental change initiatives consume resources. Providing groups in multiple settings with needed resources in the form of action packets supports implementation of initiatives that address risk factors for obesity and other chronic diseases.

# Sub article: Heart-healthy and Stroke-free: Making the Business Case to Employers and Purchasers for Preventing Heart Disease and Stroke

**Published:** 2004-03-15

**Authors:** Dyann Matson-Koffman, VA Anwuri, D Orenstein, K Shore>, L Agin, SA Garfinkel, LA Sokler>, NB Watkins, GA Mensah>

**Affiliations:** Centers for Disease Control and Prevention, Cardiovascular Health Branch, Division of Adult and Community Health, National Center for Chronic Disease Prevention and Health Promotion

The objective of this study was to highlight 2 Centers for Disease Control and Prevention (CDC) initiatives aimed at educating employers about health benefits and interventions that will have the greatest impact on preventing heart disease and stroke and reducing associated costs.

The CDC conducted a literature review and met with the National Business Group on Health to present effective interventions and promising practices for controlling heart disease and stroke and related risk factors. The CDC is also working with the American Institute of Research to develop a toolkit for states that will contain similar information.

We conducted a literature review using the Internet and ABI/Inform, LexisNexis, Medline, OVID, and PubMed databases. We identified 55 articles for health care and 22 for work site settings.

Findings suggest that the most promising interventions for improving the prevention and control of high blood pressure and high blood cholesterol in health care settings include quality care teams and protocols that follow national treatment and prevention guidelines, the use of physician and patient reminders via automated record systems, and patient education combined with quality improvement goals. In the work site setting, the most promising interventions are individual counseling and follow-up, combined with environmental supports such as health risk appraisals, wellness communications, health education classes, and access to healthy food choices and exercise facilities. On the basis of information from 9 organizations, the return-on-investment estimates ranged from $1.40 to $4.90 in savings per dollar spent for work site health management interventions.

To have the greatest impact on preventing heart disease and stroke, employers should consider individual risk-reduction counseling for high-risk employees within the context of a comprehensive systems-level approach and the most promising environmental health promotion interventions.

# Sub article: Chronic Disease Risk Factors by Ethnicity and Border Residence in Arizona: 10 Years of Behavioral Risk Factor Surveillance System Data

**Published:** 2004-03-15

**Authors:** Matt McGorray, MA Veazie

**Affiliations:** Southwest Center for Community Health Promotion, Arizona College of Public Health, Epi-Biostats

We estimated chronic disease risk factor prevalence by border county residence and Hispanic ethnicity along the U.S.-Mexico border. Hispanics living along the U.S.-Mexico border suffer from high rates of chronic diseases. Ongoing surveillance of chronic disease risk factors in this population is lacking.

We combined 10 years of Arizona Behavioral Risk Factor Surveillance System (BRFSS) data (1992–2001). The prevalence of selected risk factors was calculated for Hispanics and non-Hispanics by border county residence. After adjusting for the effects of survey design, age, and sex, we estimated the interaction between Hispanic ethnicity and residence for each risk factor in counties with cities on the border. We also mapped statewide telephone coverage among Hispanics by census tract.

Of the 20,409 respondents, 3.1%were border Hispanics, 12.3% were non-border Hispanics, 6.6% were border non-Hispanics, and 78.1% were non-border non-Hispanics. When border Hispanics are compared with non-border non-Hispanics, the age- and sex-adjusted odds ratios were 2.85 (95% Confidence Interval [CI] = 1.92, 4.23) for diabetes; 0.33 (CI = 0.25, 0.44) for self-reported good or excellent health; 1.48 (CI = 1.11, 1.98) for obesity; 0.52 (CI = 0.42, 0.64) for recommended physical activity; and 0.48 (CI = 0.38, 0.60) for current smoking.

Arizona Hispanics (represented by BRFSS survey participants) suffer from a higher prevalence of chronic disease risk factors than non-Hispanics, regardless of whether they live on the border. Results document the need to oversample on the border and to address the issue of low telephone coverage.

# Sub article: Communities Combating Chronic Disease: The Kate B. Reynolds SELF Improvement Program

**Published:** 2004-03-15

**Authors:** Maggie Sauer, JL Michener, SD Yaggy

**Affiliations:** Department of Community and Family Medicine, Division of Community Health

The objective of this study is to prevent chronic disease in at-risk populations through the development of community-based service delivery networks using community partnerships to create and sustain behavior change.

Administered by Duke University Medical Center's Department of Community and Family Medicine (CFM), on behalf of the Kate B. Reynolds Charitable Trust, the program provides funding to 16 low-income community partnerships across 21 counties in North Carolina over a 5-year period. Partners include health departments, health care organizations, schools, park and recreation departments, economic development offices, and other civic groups.

The Kate B. Reynolds Smoking, Education, Lifestyle and Fitness (SELF) Improvement Program is focused on 3 main health problems: tobacco use, inadequate nutrition, and physical inactivity. Projects are required to demonstrate effective and long-term change in the most at-risk populations through collaboration with local providers and community-based organizations as a support base for sustainability.

CFM provides technical assistance to grantees on a variety of issues, including project management and evaluation, health promotion, and service delivery.

The SELF Improvement Program is a statewide initiative that enables North Carolina communities to effect positive health changes through community-level interventions.

# Sub article: TRAILS, a School-based Walking Program

**Published:** 2004-03-15

**Authors:** William Moore, T Wilson, A Stephens, J Eichner

**Affiliations:** University of Oklahoma Prevention Research Center

The effects of a high school walking program, The Robust American Indian Lifestyle Study (TRAILS), were evaluated by measuring changes in lipid profile, aerobic capacity, and body composition. The walking program was implemented at Anadarko Public High School in southwest Oklahoma. Approximately 55% of the school's students are Native American, 34% are white, and 4% are African American. Ethnically, 7% of the students are Hispanic or Latino.

The intervention consisted of daily (Monday through Friday) self-paced walking for 35 to 40 minutes for 11 weeks. Pre- and post-intervention assessments of non-fasting lipid profile, non-fasting plasma glucose, aerobic capacity, and body composition were performed.

Twenty-five students completed the pre- and post-intervention assessments. The mean age of the students was 16.7 years (± 1.4). Sixty percent of the students were of normal weight, 4% were at risk for overweight, and 36% were overweight. The mean miles walked was 37.6 (± 10.7). Using paired t-tests, statistically significant improvements were seen in total cholesterol (174.8 to 149.4 mg/dL, P < .001); low-density lipoprotein cholesterol (106.4 to 85.1 mg/dL, P < .001); non-high-density lipoprotein cholesterol, calculated by subtracting high-density lipoprotein cholesterol from total cholesterol (128.5 to 105.9 mg/dL, P < .001); and non-fasting plasma glucose (103.2 to 82.4 mg/dL, P < .001).

A school-based walking program may have a positive impact on lipids and non-fasting plasma glucose.

# Sub article: Lessons Learned From Global Reviews of Mass Media Campaigns Designed to Reduce Smoking and Exposure to Secondhand Smoke

**Published:** 2004-03-15

**Authors:** Elizabeth Schar, KK Gutierrez

**Affiliations:** Healthcare POV

The Centers for Disease Control and Prevention (CDC) is conducting several reviews of mass media campaigns to determine the kinds of campaign elements that contribute most to success. The purpose of the reviews is to aid states and other countries in developing their own campaigns to reduce smoking and exposure to secondhand smoke. The first 2 reviews (of campaigns to promote adult smoking cessation and youth tobacco use prevention) have been completed. Preliminary findings for the third review (of campaigns to reduce exposure to secondhand smoke) are available.

Media campaigns are an effective component of a comprehensive tobacco control program. Programs must determine ways to make their limited funds work most efficiently to change attitudes and behaviors related to smoking and secondhand smoke via media campaigns.

Data and results were solicited through a variety of channels, including CDC networks, GLOBALink, and the World Health Organization. Qualitative and quantitative data as well as published and unpublished data were analyzed to understand both study results and insights into target audiences. Key measurements used to determine campaign effectiveness included changes in awareness, relevant knowledge, attitudes, and behavior.

Each campaign review produced unique findings on the following effective campaign elements: 1) a carefully targeted audience; 2) an effective message or combination of messages; 3) appropriate tone and format, including the use of emotion; 4) publicity and promotion through news media coverage; 5) sufficient media presence (reach, frequency, and duration); 6) thorough evaluation (formative, process, and outcome); and 7) synergy between the campaign and other elements of a comprehensive tobacco control program. Differences exist between the strategies and tactics needed for campaigns focused on individual change and campaigns focused on institutional or policy change.

The complete reviews illustrate key findings with examples of advertisements used in countries around the world.

Although limited by the incomplete and imperfect data collected globally, the findings provide a clear sense of direction to readers planning campaigns to encourage adult smoking cessation and youth tobacco use prevention and to reduce exposure to secondhand smoke.

# Sub article: Cost-effective, Community-based Strategies Targeting Cardiovascular Disease and Diabetes Risk Factors Among African American Women in Faith-based Environments

**Published:** 2004-03-15

**Authors:** Cheryl Taylor, L Cole, D Ferdinand, S Arline

**Affiliations:** National Black Women's Health Imperative, REACH 2010: At the Heart of New Orleans

The objective of this study was to examine the differences in heart-health behaviors of African American women in faith-based environments and the effects of faith-based interventions on behaviors.

Eliminating disparities among racial and ethnic groups and increasing quality of life and years of healthy life are the major goals of *REACH 2010* research demonstration projects. African Americans in Louisiana have alarming rates of death and disability due to heart disease and diabetes. Limited data are available on faith-based interventions among African American women.

Forty churches with memberships ranging from 100 to 15,000 each were randomized into 3 groups. The Community Health Assessment Program Survey (CHAPS), similar to the Behavioral Risk Factor Surveillance System, was conducted among 1100 African American women 18 years and older. A profile from each church was used to collect baseline aggregate data. Each group received varied doses of intervention. All churches received free annual blood pressure, body mass index, and lipid screening and counseling; screening resources were provided by community board members. Follow-up survey data were collected to determine the effects of interventions on reducing risk factors. A comparative analysis was conducted of baseline CHAPS data on 1100 women. Annual follow-up surveys are still in progress.

Participants have reported a need for chronic disease interventions that address a continuum of risk reduction, including response to emergency events such as heart attacks and strokes and referrals for rehabilitation resources. Also, the presence of respected community leaders and collaborative relationships has empowered *REACH 2010: At the Heart of New Orleans* to share resources and expand its reach to communities across the state.

# Sub article: Using Exercise for Risk Reduction in African American Breast Cancer Survivors: A Community-based Pilot Study

**Published:** 2004-03-15

**Authors:** Diane Wilson, JS Porter, G Parker, TJ Smith, J Kilpatrick

**Affiliations:** Virginia Commonwealth University School of Medicine, Internal Medicine/Massey Cancer Center

The objective of this study was to pilot test a low-impact exercise program, *Walking Counts!*, for its effect on steps walked per day and body mass index (BMI) in a population of African American breast cancer survivors.

The Massey Cancer Center in Richmond, Va, partnered with community centers to offer a walking intervention, designed by the study's primary investigator, to high-risk breast cancer survivors. More than 60% of women report weight gain after breast cancer diagnosis, increasing their risk of cancer recurrence and other co-morbidities. Few studies have tested cognitive/behavioral healthy lifestyle interventions in cancer survivors. This study was designed to measure the impact of *Walking Counts!* on steps per day, BMI, and related measures by providing skills, knowledge, and self-assessment for African American women who have had breast cancer.

An 8-week intervention was held at community locations for African American breast cancer survivors (n = 23) aged 30 to 70 years. Pedometers, a walking scheduler/tracker, and informational/motivational sessions were provided to participants to help them achieve 10,000 steps per day. Data were collected at 3 points to examine changes in walking; BMI; body fat percentage; waist, hip, and forearm circumferences; attitudes toward exercise; cancer stress; and related demographic measures.

Pre- and post-intervention impact included statistically significant increases in steps per day (*P* = .001), as well as decreases in BMI (*P* = .004), body weight (*P* = .006), percent body fat (*P* = .002), and waist (*P* = .035) and forearm (*P* = .005) circumferences. Increased positive perception of exercising was also reported. Follow-up data, including 3-month post-intervention data, will be presented to identify characteristics related to successful outcomes.

Increasing walking for exercise, without making other changes, can improve attitudes and anthropometric measures, which may help reduce risk of cancer recurrence. The high retention rate (95%), along with positive study outcomes, indicate that breast cancer survivors are motivated to improve their health habits.

# Sub article: Improving Diabetes Care With the Collaborative Model: The First North Carolina Diabetes Collaborative

**Published:** 2004-03-15

**Authors:** Marti Wolf, J Reaves, DS Porterfield, RM Carlyle, A Wang

**Affiliations:** North Carolina Diabetes Collaborative, North Carolina Primary Health Care Association

The planning, implementation, and outcomes of the North Carolina Diabetes Collaborative, modeled after the Bureau of Primary Health Care's Health Disparities Collaborative, is described.

The North Carolina Diabetes Collaborative is the result of a partnership between the North Carolina Diabetes Prevention and Control Program and the North Carolina Primary Health Care Association. An advisory council made up of strategic statewide partners complements this partnership. Fourteen teams from various health care settings across North Carolina were recruited to participate in this intervention, which focuses on improving the management of diabetes.

Participants receive technical assistance that includes learning sessions, monthly conference calls, distribution lists, and feedback on monthly reports. Teams address system-level changes and use monthly reports to track improvements in delivery of care and health outcomes. An electronic database helps the teams to identify effective interventions and to track outcomes using 8 required measures.

Improvements have been documented in the 8 required measures. Improvement has been most remarkable in the number of patients who have had their HbA1c levels checked twice per year, the number of patients with blood pressure levels below 135/85 mm Hg, the number of patients receiving foot exams, and those with low-density lipoprotein cholesterol levels below 100 mg/dL. During the first 6 months, the teams entered data on 907 patients into their disease management registries.

The North Carolina Diabetes Collaborative shows promise of increasing the quality of care for patients in participating sites. Next steps include obtaining funding for future collaboratives; expanding content area to include cardiovascular disease; incorporating feedback to improve the collaborative; and expanding the number of participants.

# Sub article: Whose Choice Is It? Understanding HIV Risk Among African American Women

**Published:** 2004-03-15

**Authors:** Elleen Yancey, LM Goodin, M Wang

**Affiliations:** Morehouse School of Medicine, Prevention Research Center

The types and prevalence of human immunodeficiency virus (HIV) risk behaviors among African American women ages 17 to 44 years were identified and an intervention was developed to reduce the risk of HIV infection by addressing culture and gender issues specific to these women.

In this intervention, we identified communities with high incidences of HIV infection and acquired immunodeficiency syndrome (AIDS) among African American women.

Before and after the intervention, an HIV Risk Reduction Survey was administered to 422 women to assess risk behavior variables. Focus groups were conducted. An intervention was developed and conducted that consisted of 7 weekly sessions. The intervention used this project's research findings and incorporated the theoretical underpinnings of 2 concepts: *Ntu* (an Africentric model of spiritual beliefs, practices, culture, and interpersonal relationships) and the Theory of Gender and Power (a social theory about sexual inequities, gender and power, and balances).

Intervention and control group comparisons before and after the intervention indicate a significant increase in HIV knowledge among women in the intervention group, based on the 12-item HIV Knowledge Scale in the Morehouse School of Medicine HIV Reduction in African American Women Survey: Intervention group mean scores pre-intervention vs post-intervention were 8.66 vs 10.01; control group mean scores pre-intervention vs post-intervention were 8.41 vs 8.42 (*P* = .01). Intention to use condoms increased among women in the intervention group but decreased among women in the control group, based on the 4-item Condom Barrier Beliefs construct (using a Likert scale of 1 to 4) in the Morehouse Survey: Intervention group mean pre-intervention vs post-intervention was 1.64 vs 1.69; control group mean pre-intervention vs post-intervention was 1.64 vs 1.61 (*P* = .05). Personal risk perceptions increased in both groups (using a 1-item Likert scale of 1 to 5), although less in the intervention group: Intervention group mean pre-intervention vs post-intervention was 1.95 vs 2.01; control group mean pre-intervention vs post-intervention was 1.96 vs 2.33 (*P* = .05).

Interventions to reduce the risk of HIV infection among African American women should help them understand relationships, facilitate increased knowledge about HIV, and support attitude and behavior changes within the context of their culture and environment. Women in this study showed an interest in seeking information on reducing their risk of HIV infection and possibly initiating steps toward behavior change. A sustained and protracted effort might be needed to help this population move from increased understanding to sustained behavior change.

# Sub article: The Spatial Analysis of CVD Mortality in a Tri-county Area of Mississippi

**Published:** 2004-03-15

**Authors:** Lei Zhang, A Penman, C Haydel, V Sutton, V Kamali, P Fos

**Affiliations:** Mississippi State Department of Health

The geographic distribution and spatial pattern of cardiovascular disease (CVD) mortality was investigated in Hinds, Rankin, and Madison counties in Mississippi from 1997 to 2000. The analysis of geographic distribution of disease mortality has an important role to play in public health and epidemiological studies.

The 1997–2000 CVD mortality data in Hinds, Rankin, and Madison counties with residential addresses were obtained from Mississippi vital statistics and geocoded to the census block groups using ArcView software. Because of the small number of CVD deaths in each block group, the geocoded records were aggregated to the related census tract. Kernel density estimator was used to calculate annual CVD mortality for each census tract. A geographically weighted regression method was used to analyze the spatial pattern of CVD mortality in the tri-county area.

For most census tracts, the changes in population density did not explain the changes in CVD death density, suggesting that the high rates were real and not an artifact of population change. The changes in CVD mortality over time were not significant for most census tracts.

These results may be useful in suggesting hypotheses for further study related to environmental factors and socioeconomic status.
